# The Golden Year? Early Intervention Yields Superior Outcomes in Chronic Pelvic Pain with Pudendal Neuralgia: A Comparative Analysis of Early vs. Delayed Treatment

**DOI:** 10.3390/life15030376

**Published:** 2025-02-27

**Authors:** Alexandru Ciudin, Albert Carrion, Rosa Regue, Alfredo Rodriguez, Eduardo Garcia-Cruz, Diana Finkelstein, Claudia Mercader, Cristian Toma, Razvan Popescu, Cristian Persu, Sergi Colom, Narcis Camps, Ramon Serrate, María José Ribal

**Affiliations:** 1 Institut de Urologia Serrate Ribal, 08017 Barcelona, Spain; albert.carrion@vallhebron.cat (A.C.); 21673rra@comb.cat (R.R.); arodriguezrodriguez@psmar.cat (A.R.); eduard.garcia.cruz@gmail.com (E.G.-C.); cmercaderb.germanstrias@gencat.cat (C.M.); scolom@bellvitgehospital.cat (S.C.); ncamps@bellvitgehospital.cat (N.C.); drserrate@drserrate.com (R.S.); mjribal@clinic.cat (M.J.R.); 2 Urology Department, Hospital Universitari de Mollet, 08100 Barcelona, Spain; 3 Urology Department, Faculty of Medicine, “Prof. Dr. Th. Burghele” Clinical Hospital, Carol Davila University of Medicine and Pharmacy, 061344 Bucharest, Romania; razvan-ionut.popescu@drd.umfcd.ro (R.P.); cristian.persu@umfcd.ro (C.P.)

**Keywords:** pudendal neuralgia, chronic pelvic pain, pudendal nerve infiltration, time to treatment

## Abstract

Background: Chronic pelvic pain (CPP) associated with pudendal neuralgia (PN) significantly impacts quality of life (QoL). Pudendal nerve infiltration is a recognized treatment, but the optimal timing of intervention remains unclear. Methods: This prospective study included 81 patients diagnosed with PN and treated with pudendal nerve infiltrations. Outcomes were assessed using the Visual Analog Scale (VAS), Spanish Pain Questionnaire (CDE–McGill), and the SF-12 health survey. Significant improvement was defined as a VAS reduction > 4 points and a QoL increase > 15 points. An ROC curve analysis identified a 13-month time-to-treatment threshold (sensitivity 78%, specificity 72%), categorizing patients into early (*n* = 27) and delayed treatment groups (*n* = 54). Results: The early treatment group showed significantly greater reductions in VAS scores (5.4 vs. 3.4 points, *p* < 0.01) and QoL improvements (18 vs. 8 points, *p* < 0.01) compared to the delayed group. Early intervention reduced reinfiltration rates (10% vs. 35%, *p* < 0.05) and decreased medication use, with 81% discontinuing gabapentin compared to 41% in the delayed group. Similar trends were observed for tryptizol (44% vs. 35%) and tramadol (74% vs. 30%). Multivariate analysis confirmed time to treatment as the strongest predictor of outcomes, with each additional month delaying treatment associated with a 0.18-point increase in final VAS scores (*p* < 0.001). Delayed treatment was linked to higher final doses of gabapentin (*p* = 0.01), dexketoprofen (*p* < 0.001), and tramadol (*p* = 0.012). Minimal complications were reported (15%, Clavien I). Conclusions: Early intervention in PN significantly improves pain, QoL, and reduces reinfiltration and medication reliance, supporting timely treatment for optimal outcomes.

## 1. Introduction

Chronic pelvic pain (CPP) is a complex condition that presents significant diagnostic and therapeutic challenges, affecting up to 24% of women worldwide at some point in their lives and profoundly impacting their quality of life [1,2,3]. The term CPP encompasses a spectrum of disorders, each with varied etiologies, including gynecological, urological, gastrointestinal, and musculoskeletal causes, making it one of the most challenging conditions to manage effectively in clinical practice [4,5].

The presentation of CPP can vary widely from patient to patient, with pain being the consistent hallmark across all cases. It often manifests as a persistent, severe, and debilitating discomfort that is frequently described as burning, aching, or stabbing sensations and which significantly disrupts daily activities and emotional well-being [3,6]. The management of CPP requires a nuanced understanding of its potential origins and pathophysiological mechanisms, which are often as varied as the symptoms themselves.

Despite the diverse etiology of CPP, a common thread in the evolution of this condition involves the eventual development of a myofascial pain component and elements of PN across virtually all phenotypes. Myofascial pain syndrome in the context of CPP involves the muscle and connective tissue and is characterized by the presence of trigger points that contribute to sustained and often referred pain patterns [7,8]. Over time, unresolved chronic pain can lead to alterations in pelvic floor muscle function, further exacerbating the discomfort and contributing to a cycle of pain and muscle spasms [3,9,10,11].

Similarly, pudendal neuralgia (PN), characterized by the entrapment or irritation of the pudendal nerve, emerges as a significant neuropathic component in many cases of CPP. This condition produces symptoms that are debilitating and often misattributed to other pelvic conditions. The pain associated with PN typically follows the distribution of the pudendal nerve, affecting the genitalia, perineum, and anal regions, and is exacerbated by sitting but relieved by standing or lying down, which are hallmark signs that help distinguish it from other types of pelvic pain [3,12,13,14].

Chronic inflammation in CPP and PN leads to micro-scarring, which complicates recovery by reducing tissue elasticity and increasing nerve compression and irritation. These micro-scars create a self-perpetuating cycle of pain and dysfunction, highlighting the need for early and effective intervention [3,12,15,16,17,18].

In the therapeutic realm, pudendal nerve infiltration has emerged as a vital treatment strategy. This procedure involves the administration of local anesthetics, with or without corticosteroids, directly around the pudendal nerve, aimed at alleviating pain by interrupting the nerve’s pain signaling. Pudendal nerve blocks are particularly effective due to their ability to target the specific site of nerve entrapment, providing significant pain relief and potentially halting the progression of nerve damage [3,19,20].

The procedure can be performed using either a transperineal or a transgluteal approach, often guided by ultrasound or fluoroscopy, to ensure accurate needle placement and effective drug delivery. This targeted approach not only offers immediate relief but also aids in diagnosing PN by confirming the nerve as the pain source when relief is achieved post-infiltration [21,22].

Given the significant impact of timely and accurate diagnosis on management outcomes, early intervention with pudendal nerve infiltration can prevent the long-term consequences of chronic pain and neuropathy, thereby maintaining functionality and improving the overall quality of life. This study emphasizes exploring the impact of time to treatment (TTT) on clinical outcomes in patients with pudendal neuralgia undergoing pudendal nerve infiltrations. Specifically, we aim to identify an optimal TTT cutoff using ROC curve analysis to differentiate early and delayed treatment groups and to evaluate its effect on pain reduction (VAS), quality of life improvement (SF-12, CDE–McGill), and medication use.

## 2. Materials and Methods

### 2.1. Study Design

This study was conducted as a cohort study with prospective inclusion of patients and a retrospective analysis of their clinical data. Patients diagnosed with PN contributing to chronic pelvic pain were systematically recruited and evaluated at the time of their diagnosis and treatment. The prospective nature of the study ensured that all patients underwent standardized diagnostic and treatment protocols, while the retrospective analysis allowed for a detailed examination of outcomes based on treatment timing.

**Figure 1:**
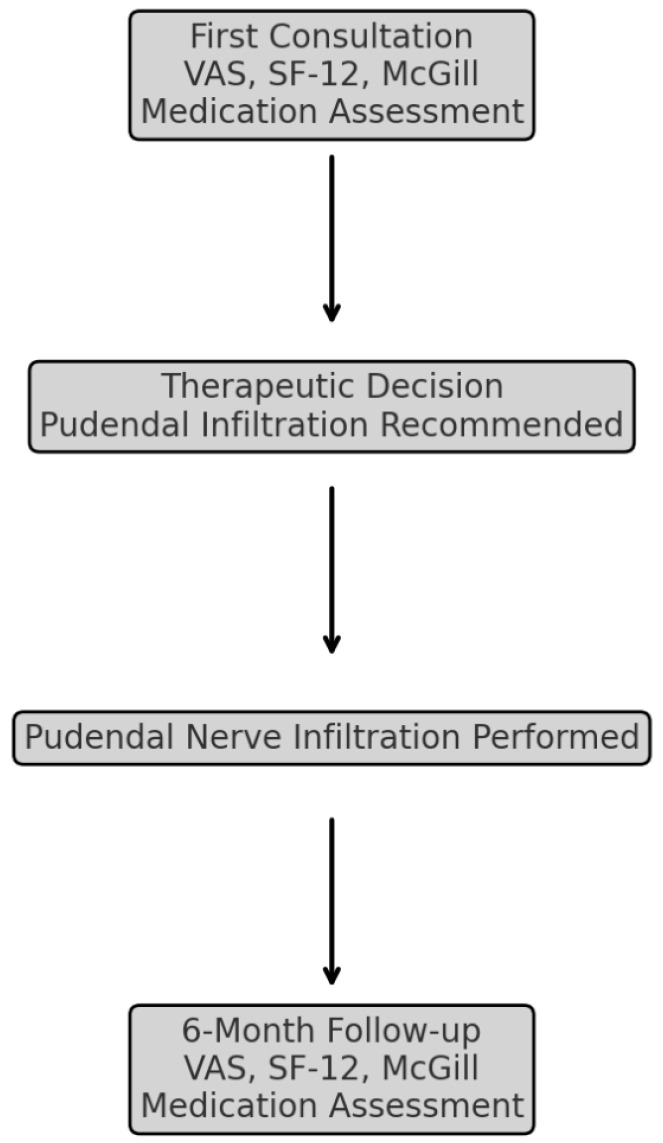
Patient flowchart: from initial consultation to six-month follow-up—a visual representation of patient inclusion, stratification, and assessments at each study phase. (VAS: Visual Analog Scale, SF-12: Short-Form Health Survey-12, McGill: Spanish Pain Questionnaire).

All patients were included in the study at the time of their first clinical evaluation, during which baseline data were systematically collected using the following validated tools (Figure 1):

- Visual Analog Scale (VAS) for pain intensity;
- SF-12 Health Survey for quality-of-life assessment;
- Spanish Pain Questionnaire (CDE–McGill) for qualitative pain descriptors and emotional impact.

The time to treatment—defined as the interval between symptom onset and pudendal nerve infiltration—was recorded for each patient during their initial evaluation. A retrospective analysis was then performed to evaluate the impact of treatment timing on outcomes, using data collected during follow-up visits.

An ROC curve analysis was employed to identify the optimal time-to-treatment cutoff point. This cutoff was used to stratify the cohort into the following two groups:

1. Early treatment.
2. Delayed treatment.

Subsequently, a comparative analysis of these two groups was performed to assess differences in pain reduction, quality of life improvements, medication use, and reinfiltration rates. This design combined the strengths of prospective data collection—ensuring consistency and completeness—with the flexibility of retrospective analysis to explore treatment outcomes and refine clinical understanding.

### 2.2. Patient Population

Patients were included in the study, and data were collected prospectively from November 2020 to March 2024, at baseline before treatment and six months after. Patients had to comply with the following inclusion and exclusion criteria to participate in this analysis.

### 2.3. Inclusion Criteria

- Diagnosed with PN based on established diagnostic criteria explained below.
- Underwent pudendal nerve infiltration as part of their treatment plan during the study period.
- Aged 18 years or older.

### 2.4. Exclusion Criteria

- Incomplete Medical Records: Cases lacking sufficient clinical, diagnostic, or follow-up data necessary for a comprehensive analysis were excluded to maintain data integrity.
- History of Significant Pelvic Surgery: Patients with prior pelvic surgeries that could substantially alter pelvic anatomy (e.g., extensive pelvic reconstruction or radical prostatectomy) were excluded to avoid confounding factors in the assessment of PN and treatment outcomes.
- Co-existing Conditions Causing Chronic Pelvic Pain: Patients with other diagnosed conditions known to independently cause chronic pelvic pain, such as interstitial cystitis, endometriosis, or sacroiliac joint dysfunction, were excluded to ensure the study focused exclusively on PN.
- Less than 18 years of age.

### 2.5. Diagnostic Criteria for PN

PN is a clinical diagnosis requiring careful evaluation of specific criteria to ensure accuracy and consistency in identifying the condition. To establish the diagnosis, the following five criteria (Nante criteria) must be assessed, with at least four out of five required for confirmation:

1. Pain in the anatomical territory of the pudendal nerve: This includes the perineum, genitals, and rectal region, reflecting the nerve’s distribution. Pain is often described as burning, stabbing, or shooting and is typically localized to these regions.
2. Pain worsened by sitting: This characteristic is highly specific to PN. Sitting increases pressure on the pudendal nerve, exacerbating symptoms, whereas standing or lying down may provide relief.
3. The patient is not woken at night by the pain: Unlike other chronic pain conditions, PN does not typically disrupt sleep, providing an important diagnostic clue to differentiate it from other causes of pelvic pain.
4. No objective sensory loss on clinical examination: Sensory testing over the pudendal nerve territory usually reveals no deficits, distinguishing the condition from other neuropathic syndromes involving sensory nerve damage.
5. Positive anesthetic pudendal nerve block: A diagnostic pudendal nerve block with local anesthetic should provide significant, temporary pain relief, confirming that the pudendal nerve is the source of the symptoms.

### 2.6. Diagnostic Algorithm

The diagnostic algorithm for PN in this study incorporated the following advanced imaging and neurophysiological tools to ensure accurate diagnosis and a comprehensive assessment of the nerve and its surrounding structures:

- Pelvic Floor Ultrasound: Used to visualize the course of the pudendal nerve and any possible entrapments or anomalies.
- 3T MRI with Neurography: Employed to provide detailed imaging of nerve paths, potential impingements, and surrounding structures.
- Neurophysiological Studies: Conducted to assess nerve function and confirm the diagnosis through sensory and motor response analyses.

### 2.7. Intervention

Pudendal nerve infiltrations were performed using a perineal approach under strict sterile conditions with patients in the lithotomy position. High-resolution ultrasound and fluoroscopic guidance were used to ensure accurate placement of the needle and effective delivery of the anesthetic mixture.

### 2.8. Technique of Pudendal Nerve Infiltration

#### 2.8.1. Preparation and Patient Positioning

Patients were prepped and draped in a sterile fashion. A detailed explanation of the procedure was provided, and verbal and written consent was obtained. Patients were then positioned in the lithotomy position on a procedure table equipped with stirrups. This position allowed for maximal exposure of the perineum and facilitated anatomical landmarks identification.

#### 2.8.2. Ultrasound Guidance

The Toshiba Arietta 65 ultrasound system (Toshiba, Tokyo, Japan), equipped with 5-1 MHz and 18-5 MHz transducers, provided high-resolution imaging for deep anatomical assessments and detailed visualization of superficial structures, ensuring diagnostic precision in pudendal neuralgia evaluation. First, the 5-1 MHz transducer was used to obtain a general overview of the pelvic structures via the perineal approach, followed by the 18-5 MHz transducer for detailed structural identification and guidance of the puncture. Ultrasound gel was applied liberally to avoid air gaps that could interfere with image clarity. The ischial tuberosity and the sacrotuberous ligament were identified as primary landmarks for the procedure. The pudendal nerve typically courses between these landmarks within Alcock’s canal. The ultrasound was used to trace the path of the pudendal nerve, ensuring accurate placement of the needle.

#### 2.8.3. Identification of Anatomical Landmarks

The use of anatomical landmarks and palpation techniques ensured accurate localization. The procedure began with the identification of the pudendal nerve pathway and associated anatomical structures. Key landmarks included the ischial spine and the sacrospinous ligament, which were palpated to confirm the trajectory of the pudendal nerve. Painful trigger points along the nerve pathway were identified through direct palpation during a vaginal or rectal examination, depending on the patient’s anatomy.

Vaginal or rectal digital examination was employed to assess the anatomy, confirm the depth of the infiltration, and ensure precise targeting of the nerve. This approach allowed for real-time feedback on the patient’s pain response, aiding in the identification of areas of heightened sensitivity. By carefully monitoring the anatomical position and adjusting the needle depth accordingly, the infiltration was delivered with precision, ensuring optimal therapeutic outcomes while minimizing the risk of complications.

#### 2.8.4. Infiltration Technique

A 22-gauge, 80 mm Pajunk SonoPlex STIM needle (Pajunk, Geisingen, Germany) was used for the infiltration. The skin and subcutaneous tissue at the chosen injection site were anesthetized using 1% lidocaine. Under continuous anatomical and ultrasound guidance, the needle was advanced towards the pudendal nerve at the level of the ischial tuberosity. Care was taken to avoid blood vessels and other neural structures.

Once the needle was in close proximity to the pudendal nerve, as confirmed by ultrasound, a test dose of saline was injected to confirm correct needle placement without resistance. Following the test injection, a mixture of long-acting local anesthetic, ropivacaine, in combination with liposoluble long-absorption corticosteroids, was slowly injected. The total volume injected did not exceed 13 mL to minimize the risk of tissue distortion and potential nerve damage. The combination was 10 mL of 2% ropivacaine with 3 mL of triamcinolone acetonide at 40 mg/mL concentration.

#### 2.8.5. Post-Procedure Care and Observation

After the procedure, patients were monitored for any immediate adverse reactions or complications such as hematoma, infection, or allergic reaction. If no adverse effect was reported in the first 30 min, patients were discharged and advised to avoid strenuous activities for 24 to 48 h.

### 2.9. Data Collection

Collected data included demographic information, duration of symptoms before intervention, details of the infiltration procedure, and medical treatments employed by each patient. Pain scores were measured using the Visual Analog Scale (VAS) and CDE–McGill questionnaire, and quality of life was assessed with the SF-12 health survey, both before and 6 months after the treatment.

### 2.10. Outcome Measures

1. Primary Objective:

The main objective of this study is to evaluate the impact of time to treatment (TTT) on clinical outcomes in patients with pudendal neuralgia undergoing pudendal nerve infiltrations. Specifically, we aim to determine an optimal TTT cutoff using ROC curve analysis to differentiate early and delayed treatment groups and to assess its effect on pain reduction (VAS).

2.  Secondary Objectives:
  - Quality of Life Assessment: To compare changes in SF-12 and CDE–McGill questionnaire scores between early and delayed treatment groups.
  - Medication Reduction Analysis: To evaluate differences in the discontinuation or dose reduction of gabapentin, tryptizol, dexketoprofen, and tramadol between treatment groups.
  - Complication and Safety Profile: To document adverse events associated with pudendal nerve infiltrations, categorized using the Clavien–Dindo classification.
  - Predictors of Clinical Outcomes: To identify patient-related factors, such as baseline pain severity (VAS), age, and gender, that may influence post-treatment outcomes using multivariate regression analysis.
  - Validation of Time to Treatment as an Independent Predictor: To confirm whether the identified 13-month cutoff serves as an independent predictor of superior pain relief and quality of life improvement.

### 2.11. Statistical Analysis

Descriptive statistics summarized the demographic and clinical characteristics of the study population. Outcomes were analyzed using the Student’s *t*-test or the Mann–Whitney U test, as appropriate. Multivariate regression analysis was used to identify predictors of outcomes, adjusting for potential confounders. All analyses were performed using the statistical software SPSS 16.0.

## 3. Results

### 3.1. Study Population Characteristics

A total of 81 patients were included in the study, with a predominance of females (64%). The average age of participants was 47 years (SD ± 11.6). The baseline characteristics are detailed in Table 1, which includes demographic data and the initial severity of symptoms.

### 3.2. Intervention Outcomes

#### ROC Curve Analysis

**Figure 2:**
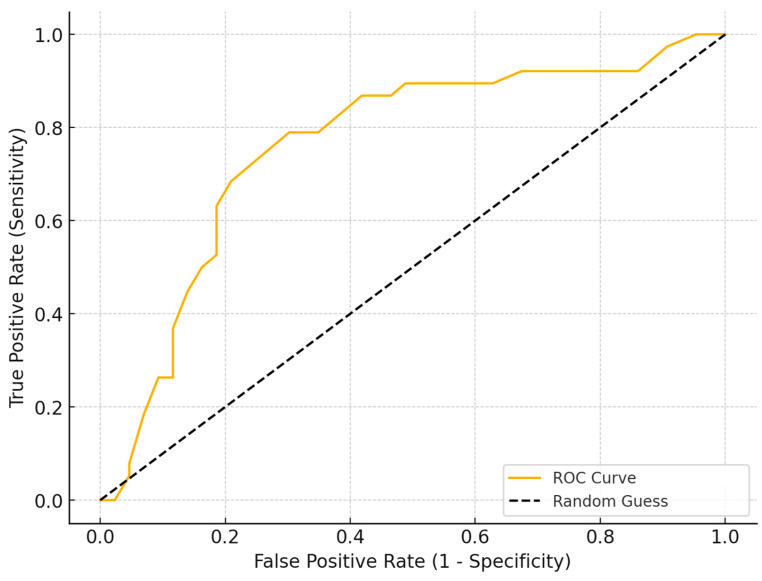
ROC curve analysis to determine the optimal time-to-treatment cutoff for significant pain reduction (VAS > 4 points). The analysis identified a threshold of 13 months, with a sensitivity of 78% and specificity of 72%, distinguishing the early and delayed treatment groups. (ROC: Receiver Operating Characteristic, VAS: Visual Analog Scale).

The ROC curve analysis was conducted to determine the optimal cutoff for time to treatment in predicting significant improvement, defined as a reduction in VAS scores greater than four points. The analysis identified a threshold of 13 months as the optimal cutoff, with a sensitivity of 78% and specificity of 72%. Based on this analysis, two groups of patients were defined: the early treatment group (less than 13 months—27 patients) and the delayed treatment group (more than 13 months—54 patients) (Figure 2).

### 3.3. Pain Relief and Quality of Life

#### VAS Evolution

Significant reductions in pain intensity were observed, as measured by the Visual Analog Scale (VAS). Patients in the early treatment group, who received intervention within 12 months of symptom onset, showed a substantial reduction in VAS scores from a baseline average of 8.6 (SD ± 0.9) to 3.2 (SD ± 1.3). In contrast, the delayed treatment group exhibited a more modest decrease, with VAS scores declining from an average of 9.0 (SD ± 1.0) to 5.1 (SD ± 1.6) (Table 2).

### 3.4. Quality of Life Evolution

Improvements in quality of life, evaluated through the SF-12 health survey, were similarly more pronounced in the early treatment group. Their scores improved markedly from a baseline of 31.2 (SD ± 5.4) to 48.5 (SD ± 4.0), compared to the delayed treatment group, where scores increased from 30.4 (SD ± 5.7) to 40.3 (SD ± 6.2). These findings highlight the enhanced benefits of timely intervention in reducing pain and improving the overall quality of life for patients with PN.

### 3.5. CDE–McGill Questionnaire Outcomes

The CDE–McGill Pain Questionnaire revealed significant reductions in overall pain scores. Patients in the early treatment group showed a substantial decrease, with scores dropping from a mean of 75.8 (SD ± 9.0) to 29.8 (SD ± 6.7). The delayed treatment group also experienced a reduction, though less pronounced, with scores declining from a mean of 76.2 (SD ± 9.3) to 49.3 (SD ± 9.1). These results highlight the greater effectiveness of early intervention in alleviating pain as measured by the CDE–McGill scale.

### 3.6. Qualitative Changes in the CDE–McGill Questionnaire

Analysis of the CDE–McGill Pain Questionnaire revealed significant qualitative improvements in the descriptors of pain experienced by patients following treatment. In the early intervention group, the proportion of patients reporting severe neuropathic descriptors, such as “burning”, “shooting”, and “electric shocks”, decreased from 78% (21 out of 27) pre-treatment to 15% (4 out of 27) post-treatment. Conversely, terms indicating milder pain, such as “tingling” or “aching”, increased from 11% (3 out of 27) to 63% (17 out of 27). Emotional descriptors like “distressing” and “unbearable” were also significantly reduced, with only 7% (2 out of 27) of patients using such terms post-treatment compared to 67% (18 out of 27) pre-treatment.

In the delayed treatment group, qualitative improvements were less pronounced. Severe pain descriptors decreased from 74% (40 out of 54) to 48% (26 out of 54), while mild pain descriptors increased from 15% (8 out of 54) to 30% (16 out of 54). Emotional descriptors showed similar trends, with 60% (32 out of 54) of patients reporting “distressing” or “unbearable” pain pre-treatment, reducing to 37% (20 out of 54) post-treatment.

These results highlight the transformative effect of early intervention, not only reducing pain intensity but also significantly altering the quality of pain experienced by patients, further underscoring the broader benefits of timely treatment.

### 3.7. Time to Treatment as a Significant Predictor of Post-Treatment VAS Scores and Reduction in Medication Doses

Multivariate regression analysis identified time to treatment as a significant predictor of post-treatment VAS scores. Each additional month of delay in treatment was associated with an average increase of 0.18 points in the final VAS score (*p* < 0.001). Initial VAS severity was also strongly associated with final VAS scores, with higher baseline scores leading to higher post-treatment values (*p* < 0.001).

The treatment group played a critical role, with patients in the early treatment group showing significantly lower final VAS scores compared to those in the delayed treatment group (*p* < 0.001). Similarly, time to treatment was significantly associated with reductions in medication doses. Patients with delayed treatment required higher final doses of gabapentin (*p* = 0.01) and dexketoprofen (*p* < 0.001), reflecting reduced effectiveness in achieving symptom control. For tramadol, a similar trend was observed, with delayed treatment linked to smaller reductions in dose (*p* = 0.012). Although gender was not statistically significant (*p* = 0.062), male patients tended to have slightly lower final VAS scores than female patients. Age, on the other hand, did not significantly influence final VAS scores (*p* = 0.754).

### 3.8. Additional Treatments

A pronounced decrease in the need for additional pain medications was noted in the early treatment group compared to the delayed treatment group (Appendix A). Specifically, in the early treatment group, 81% of patients stopped using gabapentin, reducing their average dose from 878 mg to 77.78 mg per patient, compared to only 41% of patients in the delayed treatment group, where the dose decreased from 900 mg to 383.33 mg per patient. Similar trends were observed for tryptizol, with 44% of early-treated patients discontinuing its use and the average dose decreasing from 39 mg to 11.11 mg per patient, while in the delayed group, only 35% discontinued use, with a smaller reduction from 38 mg to 13.70 mg per patient. For dexketoprofen, 74% of patients in the early group stopped its use, with an average dose reduction from 67 mg to 8.33 mg per patient, compared to the delayed group, where only 26% discontinued its use and the average dose decreased from 64 mg to 38.43 mg per patient. Lastly, for tramadol, 74% of early-treated patients discontinued use, reducing the average dose from 102 mg to 12.96 mg per patient, whereas in the delayed group, only 30% stopped using it, with a reduction from 106 mg to 72.22 mg per patient (Table 3).

These findings underscore the significant advantage of early intervention in reducing the need for and the average dose of pain medications, highlighting the broader benefits of timely treatment in managing PN.

Subgroup analyses revealed significant differences in dose adjustments between females and males (Table 4), particularly among patients with high initial VAS scores (≥8). Females showed a lesser reduction in gabapentin dosage, from 912.77 mg to 306.38 mg, compared to males, whose dosage decreased from 855.56 mg to 188.89 mg (*p* = 0.03). Similarly, females reduced their tryptizol dosage from 38.3 mg to 13.62 mg, while males reduced from 39.07 mg to 12.96 mg, though this difference was not statistically significant (*p* = 0.08).

For dexketoprofen, males with high VAS scores exhibited greater reductions, from 63.89 mg to 21.30 mg, compared to females, who reduced from 64.36 mg to 32.98 mg (*p* = 0.04). In contrast, males had less pronounced reductions in tramadol dosage, from 98.15 mg to 51.85 mg, whereas females reduced from 108.51 mg to 55.32 mg (*p* = 0.02). These findings suggest that while both genders benefit from early intervention, males tend to experience greater reductions in certain medications, particularly dexketoprofen and gabapentin, while females exhibit more pronounced decreases in tramadol dosage (Table 5).

### 3.9. Patient Satisfaction and Follow-Up

High levels of patient satisfaction were noted, particularly in the early treatment group. At the six-month follow-up, 11% of patients in the early treatment group required re-infiltration, compared to 35% in the delayed treatment group, indicating the sustained efficacy of the initial intervention. A Chi-square test was performed to evaluate this difference in re-infiltration rates, demonstrating statistical significance (*p* < 0.05). Furthermore, no significant adverse events were observed during the study. The only reported complications were classified as Clavien–Dindo Grade I, consisting of transient pain along the infiltration trajectory in a small subset of patients (12 patients—15%, 4 in the early group and 8 in the delayed group—15% in each group), which resolved within a few days and was effectively managed with standard analgesic therapy. These findings highlight not only the long-term benefits of early intervention in PN but also its safety and tolerability, further reinforcing its role as a first-line approach in managing this challenging condition.

## 4. Discussion

Pudendal neuralgia is a complex neuropathic pain condition characterized by chronic pelvic pain, often leading to significant functional impairment and reduced quality of life. The management of PN remains challenging due to its multifactorial etiology, the variability in patient responses to treatment, and the lack of consensus on optimal treatment timing [3,23].

Our study is the first to definitively demonstrate the critical importance of early intervention in PN. Patients who received treatment within 13 months of symptom onset showed significantly better outcomes in pain reduction, as measured by the Visual Analog Scale (VAS), and improved quality of life compared to those with delayed treatment. These findings are groundbreaking in the context of the existing literature, which has largely focused on the efficacy of various treatments but has not emphasized the timing of intervention [24,25,26]. The 13-month cutoff for defining early vs. delayed treatment was determined based on an ROC curve analysis, which identified this threshold as the optimal balance between sensitivity (78%) and specificity (72%) in predicting significant pain reduction (VAS > 4 points). This data-driven approach ensures that the classification of treatment timing is statistically robust rather than arbitrarily chosen. Furthermore, while there is limited literature specifically defining optimal time-to-treatment thresholds for pudendal neuralgia, studies on other neuropathic pain conditions suggest that prolonged delays in intervention lead to central sensitization and reduced treatment efficacy [9,27,28,29,30,31,32,33]. Research on chronic pain syndromes supports the concept that earlier intervention is associated with better outcomes [3,34,35,36], reinforcing the clinical validity of our cutoff. Future studies should explore whether this threshold remains consistent across different patient populations and treatment modalities, further refining the definition of timely intervention in pudendal neuralgia.

Several studies have explored the effectiveness of pudendal nerve infiltration and surgical decompression, reporting significant improvements in pain and function [3,19,20,25,37,38]. However, these studies did not assess the impact of treatment timing. Our results highlight that timely intervention can prevent symptom progression, central sensitization, and potentially irreversible neuropathic changes, aligning with broader principles of early management in neuropathic conditions [2,3].

The findings of this study highlight the overall efficiency of treatment in managing PN, with pudendal nerve infiltrations proving to be a highly effective intervention. These infiltrations not only led to substantial pain reduction, as evidenced by significant decreases in the VAS and CDE–McGill scores, but also facilitated remarkable improvements in quality of life, reflected in the SF-12 health survey scores. The ability of this treatment to achieve sustained pain relief while reducing dependence on additional medications highlights its dual benefit in enhancing patient outcomes and minimizing treatment burdens. These results reaffirm the critical role of pudendal nerve infiltrations as a cornerstone in the management of this debilitating condition.

Some studies have suggested that the use of corticosteroids does not provide additional benefits in pudendal nerve infiltrations. However, our study, despite its limitations of being non-randomized and lacking a control group, supports the efficacy of combining local anesthetics with slow-absorption liposoluble corticosteroids. This combination appears to enhance pain relief and improve quality of life, potentially due to the prolonged anti-inflammatory effects of the corticosteroids. These findings highlight the importance of further randomized controlled trials to confirm the added value of corticosteroids in pudendal nerve infiltrations and to optimize treatment protocols for PN.

Our study demonstrates that early intervention in PN significantly reduces the necessity for medications such as gabapentin, tryptizol, dexketoprofen, and tramadol, with a notable proportion of patients discontinuing these drugs entirely. This reduction not only lessens the burden of long-term pharmacotherapy but also minimizes the risk of adverse effects associated with these medications. These findings align with the existing literature advocating for multimodal early treatment strategies. For instance, a systematic review highlighted the efficacy of combined therapeutic approaches in managing PN, emphasizing the benefits of early and comprehensive treatment plans [23]. Similarly, the European Association of Urology’s guidelines on chronic pelvic pain recommend early multimodal interventions to optimize patient outcomes. Our results further support these recommendations, underscoring the importance of prompt and combined therapeutic strategies in effectively reducing pain and enhancing the quality of life for patients with PN [3,23].

The superior outcomes observed in the early treatment group may be attributed to neurophysiological mechanisms involved in pain chronification. Prolonged nociceptive input from an entrapped or irritated pudendal nerve can lead to central sensitization, a process in which repeated pain signaling amplifies neuronal excitability within the spinal cord and brain, resulting in persistent pain even after the initial peripheral trigger subsides [27,29]. Early intervention with pudendal nerve infiltrations likely disrupts this cycle before long-term neuroplastic changes occur, preventing maladaptive central reorganization. Furthermore, delayed treatment may allow for the progression of neuroinflammatory processes, leading to microglial activation, increased excitability of dorsal horn neurons, and a heightened pain response [28,30,39]. These mechanisms align with previous findings in neuropathic pain research, where earlier therapeutic intervention is associated with better long-term outcomes [3,31,40]. By mitigating central sensitization and neuroinflammatory changes, timely pudendal nerve infiltration appears to preserve normal pain modulation pathways, leading to more pronounced and sustained symptom relief compared to delayed treatment.

Comparing our findings to international studies, it is evident that while various interventions—ranging from physical therapy to nerve blocks—have been shown to improve PN symptoms, none have specifically addressed the role of treatment timing in optimizing outcomes [23,41]. Our study fills this critical gap in the literature, providing robust evidence that early intervention is a key determinant of treatment success.

Subgroup analyses revealed that while both genders benefit from early intervention, males tend to experience greater reductions in certain medications, particularly dexketoprofen and gabapentin, while females exhibit more pronounced decreases in tramadol dosage. Hormonal influences and variations in drug metabolism could contribute to these discrepancies, with females potentially exhibiting greater sensitivity to opioids, leading to more significant reductions in tramadol use. Conversely, the greater reductions in gabapentin and dexketoprofen among males may indicate differences in neuropathic pain processing or tolerability. However, age did not significantly influence outcomes for pain reduction but did play a role in the final doses of certain medications. These findings suggest that baseline pain severity and gender are important predictors of treatment response. An important consideration is the long-term impact of pharmacotherapy. Chronic use of neuropathic pain medications, including gabapentin, tricyclic antidepressants, and opioids, is associated with risks such as cognitive impairment, dependency, and metabolic side effects [3,32,33]. By demonstrating significant medication tapering in the early treatment group, our findings emphasize the clinical advantage of timely intervention in reducing long-term exposure to these risks. Future studies should further explore the long-term effects of pharmacological management versus interventional strategies to optimize treatment pathways for patients with chronic pelvic pain.

Our multivariate regression analysis highlights the critical role of baseline clinical variables in determining therapeutic outcomes for PN. While initial VAS severity emerged as a significant predictor of post-treatment VAS scores (*p* < 0.001), its influence on final medication dose adjustments was less pronounced. Instead, time to treatment was identified as a key determinant of final medication doses. Patients in the delayed treatment group required significantly higher final doses of gabapentin (*p* = 0.01) and dexketoprofen (*p* < 0.001), underscoring the detrimental effects of postponing intervention and further supporting the benefits of early treatment.

These findings collectively emphasize the value of incorporating patient-specific variables, such as symptom severity, treatment timing, and demographic factors, into personalized management strategies to optimize therapeutic outcomes. Early intervention remains critical for achieving greater reductions in pain and minimizing the need for long-term medication dependence [42].

Despite the strengths of our study, including its prospective cohort design, comprehensive analysis of treatment timing, medication usage, and patient-reported outcomes, certain limitations should be acknowledged. Although the prospective nature minimizes recall bias and enhances data reliability, the sample size, while robust, warrants validation through larger multicenter studies to ensure generalizability. Additionally, the absence of a control group and randomization limits the ability to draw definitive causal inferences.

One of the main limitations of this study is the absence of blinding, both for patients and evaluators, which may introduce bias in the assessment of subjective outcomes such as VAS and SF-12. Patient expectations and psychological factors can influence self-reported pain and quality of life scores, potentially exaggerating the perceived benefits of early intervention. Similarly, evaluators aware of treatment timing might unconsciously interpret post-treatment improvements more favorably. While objective measures, such as medication dose reduction, provide additional support for the efficacy of early treatment, future studies should aim to incorporate blinding strategies—such as independent outcome assessors or placebo-controlled designs—to minimize potential bias and strengthen the reliability of findings.

Although this study employed multivariate regression analysis to adjust for key clinical predictors, other potential confounding factors may have influenced treatment outcomes. To minimize potential bias, our multivariate regression model included key clinical covariates such as baseline pain severity (VAS), gender, and symptom duration. These variables were selected based on their known influence on pain perception and treatment response in chronic pain syndromes. By incorporating them into the model, we ensured that the observed impact of time to treatment on outcomes was independent of these factors. Our analysis confirmed that time to treatment remained the strongest predictor of post-treatment VAS scores and medication reduction, even after adjusting for these confounders. However, while our model accounts for major known confounders, residual bias cannot be entirely excluded. Psychological distress, including anxiety and depression, has been shown to modulate pain perception [43,44] and may have affected individual responses to pudendal nerve infiltrations. Additionally, socioeconomic status could impact access to early diagnosis and treatment [34], potentially contributing to disparities in outcomes between early and delayed treatment groups. Moreover, concurrent treatments, such as physical therapy and lifestyle modifications, were not systematically analyzed in this study but could have played a role in patient recovery. Future research should incorporate these variables into predictive models to provide a more comprehensive understanding of the factors influencing treatment success while also focusing on confirming these findings through randomized controlled trials, further exploring the mechanisms underlying the observed benefits of early intervention, and refining protocols for the early identification and management of pudendal neuralgia. Expanding diagnostic algorithms and incorporating advanced imaging and neurophysiological studies could also enhance diagnostic precision and treatment outcomes.

## 5. Conclusions

Our study is the first to establish that early intervention is essential in the management of PN. Timely treatment not only reduces pain and improves quality of life but also minimizes medication dependency and its associated risks. These results set a new benchmark in the treatment of PN, highlighting the importance of early diagnosis and intervention to optimize patient outcomes and enhance the efficiency of healthcare resource utilization.

## Figures and Tables

**Table 1:** Baseline characteristics of study participants—demographic and clinical data comparison between the early and delayed treatment groups. (SD: Standard Deviation, VAS: Visual Analog Scale, SF-12: Short-Form Health Survey-12, CDE–McGill: Spanish Pain Questionnaire).

Characteristic	Total (*n* = 81) Mean ± SD	Early Treatment (*n*) Mean ± SD	Delayed Treatment (*n*) Mean ± SD	*p*-Value
Age (years)	47.3 (±11.6)	49.1 (±9.3)	46.5 (±12.6)	0.29
Gender—female (%)	64.2%	48.1%	64.8%	
Gender—male (%)	35.8%	51.9%	35.2%	
Time since symptoms onset (months)	21.8 (±10.1)	10.6 (±1.9)	27.4 (±7.6)	<0.01
VAS score at baseline	8.9 (±1.0)	8.6 (±0.9)	9.0 (±1.0)	0.08
SF-12 score at baseline	30.7 (±5.4)	31.2 (±5.0)	30.4 (±5.7)	0.45
CDE–McGill score at baseline	76.1 (±9.0)	75.8 (±8.7)	76.2 (±9.2)	0.84

**Table 2:** Pain and Quality of Life Evolution—Comparison of pre- and post-treatment VAS, SF-12, and CDE–McGill scores (VAS: Visual Analog Scale, SF-12: Short-Form Health Survey-12, CDE–McGill: Spanish Pain Questionnaire, SD: Standard Deviation, *p*-value: Probability Value).

Variable	Pre-Treatment Mean ± SD	Post-Treatment Mean ± SD	*p*-Value (Pre vs. Post)	*p*-Value (Early vs. Delayed Post)
VAS score				
Early treatment	8.6 (±0.9)	3.2 (±1.3)	<0.000	0.01
Delayed treatment	9.0 (±1.0)	5.1 (±1.6)	<0.000	
SF-12 score				
Early treatment	31 (±5)	49 (±4)	<0.000	0.02
Delayed treatment	30 (±5)	40 (±6)	<0.000	
CDE–McGill score				
Early treatment	76 (±9)	30 (±7)	<0.000	0.01
Delayed treatment	76 (±9)	49 (±9)	<0.000	

**Table 3:** Multivariate regression for predicting final VAS (VAS: Visual Analog Scale, CDE–McGill: Spanish Pain Questionnaire, SF-12: Short-Form Health Survey-12, R^2^: Coefficient of Determination, *p*-value: Probability Value).

Predictor	Coefficient (B)	95% Confidence Interval	*p*-Value
Constant	−3.31	−8.31 to 1.70	0.192
Early treatment	−1.92	−2.80 to −1.04	<0.001
CDE–McGill baseline	0.02	−0.017 to 0.056	0.285
Initial VAS	0.61	0.26 to 0.96	0.001
Age	0.0014	−0.027 to 0.030	0.924
Gender (1 = Male)	−0.26	−0.96 to 0.44	0.460
SF-12 baseline	0.0025	−0.059 to 0.064	0.937
SF-12 post-treatment	0.0346	−0.026 to 0.095	0.257

R^2^ = 0.388; Adjusted R^2^ = 0.329; *p* (model) < 0.001.

**Table 4:** Subgroup analysis—medication tapering differences by gender and baseline pain severity. (VAS: Visual Analog Scale, mg: Milligrams, SD: Standard Deviation, *p*-value: Probability Value).

Subgroup	Patient Count	Gabapentin (Initial)	Gabapentin (Final)	Tryptizol (Initial)	Tryptizol (Final)	Dexketoprofen (Initial)	Dexketoprofen (Final)	Tramadol (Initial)	Tramadol (Final)
Female, moderate VAS	5	960 ± 100	600 ± 80	42 ± 6	10 ± 4	70 ± 12	35 ± 8	100 ± 15	40 ± 9
Female, high VAS	47	912 ± 110	306 ± 75	38 ± 8	13 ± 5	64 ± 10	32 ± 7	108 ± 14	55 ± 10
Male, moderate VAS	2	750 ± 90	150 ± 60	25 ± 5	0 ± 0	75 ± 10	0 ± 0	100 ± 10	25 ± 5
Male, high VAS	27	855 ± 105	188 ± 70	39 ± 7	12 ± 5	63 ± 12	21 ± 6	98 ± 12	51 ± 8

**Table 5:** Average dose per patient by medication—comparison of medication use pre- and post-treatment in both groups. (mg: Milligrams, SD: Standard Deviation, *p*-value: Probability Value).

Medication	Group	Patients Pre-Treatment (*n*)	Patients Who Stopped Using (*n*)	Average Dose Before Treatment (mg)	Average Dose After Treatment (mg)	Reduction in Average Dose (mg)
Gabapentin	Early	27	22	877.78 ± 210.4	77.78 ± 32.5	800.00 ± 288.2
Gabapentin	Delayed	54	22	900.00 ± 230.1	383.33 ± 125.6	516.67 ± 305.1
Tryptizol	Early	27	12	38.70 ± 12.1	11.11 ± 4.7	27.59 ± 11.5
Tryptizol	Delayed	54	19	38.33 ± 10.9	13.70 ± 5.3	24.63 ± 9.5
Dexketoprofen	Early	27	20	66.67 ± 15.3	8.33 ± 3.9	58.33 ± 20.8
Dexketoprofen	Delayed	54	14	63.89 ± 18.7	38.43 ± 11.5	25.46 ± 17.3
Tramadol	Early	27	20	101.85 ± 22.6	12.96 ± 5.1	88.89 ± 19.4
Tramadol	Delayed	54	16	105.56 ± 24.9	72.22 ± 19.8	33.33 ± 18.6

## Data Availability

The data are not available due to the Spanish Organic Law on Data Protection.

## Appendix A

**Table A1:** Medication evolution with treatment.

Patient ID	Group	Gabapentin (Initial mg)	Gabapentin (Final mg)	Tryptizol (Initial mg)	Tryptizol (Final mg)	Dexketoprofen (Initial mg)	Dexketoprofen (Final mg)	Tramadol (Initial mg)	Tramadol (Final mg)
1	Early	600	300	35	20	50	0	50	0
2	Early	900	0	25	0	75	0	150	0
3	Early	1200	0	50	0	75	0	100	50
4	Early	900	0	35	20	50	25	150	0
5	Early	600	0	50	0	75	0	100	0
6	Early	1200	0	25	0	75	0	50	0
7	Early	600	300	50	30	75	25	150	0
8	Early	900	0	25	0	50	50	50	0
9	Early	1200	0	35	20	75	25	100	50
10	Early	600	0	50	30	50	0	150	0
11	Early	900	0	25	0	75	0	100	50
12	Early	1200	600	50	0	75	0	50	0
13	Early	600	0	35	10	75	50	100	0
14	Early	900	0	50	0	50	0	150	50
15	Early	1200	600	25	0	75	0	50	0
16	Early	600	0	50	10	75	0	100	50
17	Early	900	0	35	10	50	0	50	0
18	Early	1200	0	50	20	75	0	150	50
19	Early	600	0	25	0	75	25	50	0
20	Early	900	0	50	20	50	0	150	50
21	Early	600	300	35	20	50	25	50	0
22	Early	900	0	25	0	75	0	150	0
23	Early	1200	0	50	30	75	0	100	0
24	Early	900	0	35	20	50	0	150	0
25	Early	600	0	50	10	75	0	100	0
26	Early	1200	0	25	0	75	0	50	0
27	Early	600	0	50	30	75	0	150	0
28	Delayed	600	300	35	20	50	25	50	0
29	Delayed	900	600	25	10	75	50	150	0
30	Delayed	1200	900	50	30	75	0	100	150
31	Delayed	900	0	35	0	50	50	150	0
32	Delayed	600	300	50	20	50	25	100	50
33	Delayed	1200	900	25	10	75	75	50	100
34	Delayed	900	600	50	30	50	50	150	150
35	Delayed	600	0	25	0	75	25	50	50
36	Delayed	1200	900	50	20	75	75	150	0
37	Delayed	900	600	35	20	50	50	100	150
38	Delayed	600	0	50	20	50	0	150	50
39	Delayed	1200	900	25	10	75	75	50	0
40	Delayed	900	600	50	30	75	50	150	150
41	Delayed	600	0	35	0	50	0	100	50
42	Delayed	1200	900	25	10	75	75	50	0
43	Delayed	900	0	50	30	50	50	150	150
44	Delayed	600	300	25	0	75	0	50	50
45	Delayed	1200	900	50	20	75	0	150	0
46	Delayed	900	0	35	20	50	50	100	150
47	Delayed	600	0	50	20	50	25	150	50
48	Delayed	1200	900	25	10	75	75	50	100
49	Delayed	900	600	50	30	75	50	150	150
50	Delayed	600	0	35	0	50	0	100	50
51	Delayed	1200	0	25	0	75	75	50	100
52	Delayed	900	0	50	30	50	50	150	0
53	Delayed	600	0	25	10	75	25	50	50
54	Delayed	1200	900	50	0	75	75	150	100
55	Delayed	600	300	25	0	75	25	50	0
56	Delayed	1200	900	50	20	75	75	150	100
57	Delayed	900	0	35	20	50	50	100	150
58	Delayed	600	300	50	20	50	25	150	50
59	Delayed	1200	0	25	0	75	75	50	100
60	Delayed	900	600	50	30	75	50	150	150
61	Delayed	600	0	35	20	50	25	100	0
62	Delayed	1200	0	25	0	75	0	50	0
63	Delayed	900	0	50	30	50	0	150	150
64	Delayed	600	0	25	0	75	0	50	0
65	Delayed	1200	900	50	0	75	0	150	0
66	Delayed	900	600	35	20	50	0	100	150
67	Delayed	600	300	50	20	50	25	150	0
68	Delayed	1200	0	25	0	75	75	50	0
69	Delayed	900	600	50	0	75	0	150	0
70	Delayed	600	0	35	20	50	25	100	50
71	Delayed	1200	900	25	0	75	75	50	100
72	Delayed	900	600	50	30	50	50	150	150
73	Delayed	600	300	25	0	75	0	50	50
74	Delayed	1200	0	50	20	75	75	150	100
75	Delayed	900	600	35	20	50	50	100	150
76	Delayed	600	300	50	20	50	0	150	50
77	Delayed	1200	900	25	0	75	75	50	100
78	Delayed	900	0	50	30	75	50	150	150
79	Delayed	600	0	35	20	50	25	100	50
80	Delayed	1200	900	25	0	75	75	50	100
81	Delayed	900	600	50	0	50	50	150	150

## References

[1] Passavanti M.B., Pota V., Sansone P., Aurilio C., De Nardis L., Pace M.C. (2017). Chronic Pelvic Pain: Assessment, Evaluation, and Objectivation. Pain Res. Treat..

[2] Engeler D.S., Baranowski A.P., Dinis-Oliveira P., Elneil S., Hughes J., Messelink E.J., Van Ophoven A., Williams A.C. (2013). The 2013 EAU Guidelines on Chronic Pelvic Pain: Is Management of Chronic Pelvic Pain a Habit, a Philosophy, or a Science? 10 Years of Development. Eur. Urol..

[3] Engeler D., Baranowski A.P., Berghmans B., Birch J., Borovicka J., Cottrell A.M., Dütschler J., Dinis-Oliveira P., Elneil S., Flink I. EAU Guidelines on Chronic Pelvic Pain. https://uroweb.org/guidelines/chronic-pelvic-pain.

[4] Reiter R.C. (1998). Evidence-Based Management of Chronic Pelvic Pain. Clin. Obstet. Gynecol..

[5] Juganavar A., Joshi K.S. (2022). Chronic Pelvic Pain: A Comprehensive Review. Cureus.

[6] Pinto L., Soutinho M., Fernandes M.C., Táboas M.I., Leal J., Tomé S., Moreira J., Zão A. (2024). Chronic Primary Pelvic Pain Syndromes in Women: A Comprehensive Review. Cureus.

[7] Ross V., Detterman C., Hallisey A. (2021). Myofascial Pelvic Pain: An Overlooked and Treatable Cause of Chronic Pelvic Pain. J. Midwifery Women Health.

[8] Pastore E.A., Katzman W.B. (2012). Recognizing Myofascial Pelvic Pain in the Female Patient with Chronic Pelvic Pain. J. Obstet. Gynecol. Neonatal Nurs..

[9] Won H.R., Abbott J. (2010). Optimal Management of Chronic Cyclical Pelvic Pain: An Evidence-Based and Pragmatic Approach. Int. J. Women Health.

[10] Aredo J.V., Heyrana K.J., Karp B.I., Shah J.P., Stratton P. (2017). Relating Chronic Pelvic Pain and Endometriosis to Signs of Sensitization and Myofascial Pain and Dysfunction. Semin. Reprod. Med..

[11] Bonder J.H., Chi M., Rispoli L. (2017). Myofascial Pelvic Pain and Related Disorders. Phys. Med. Rehabil. Clin. N. Am..

[12] Yoon S.J., Gómez-Hoyos J., Márquez-Arabia W.H., Martin H.D. (2018). Pudendal Nerve Neuralgia/Entrapment. Posterior Hip Disorders: Clinical Evaluation and Management.

[13] Antolak S.J. (2008). Pudendal Neuralgia. Genitourinary Pain and Inflammation.

[14] Khoder W., Hale D. (2014). Pudendal Neuralgia. Obstet. Gynecol. Clin. N. Am..

[15] Quaghebeur J., Wyndaele J.J., De Wachter S. (2017). Pain Areas and Mechanosensitivity in Patients with Chronic Pelvic Pain Syndrome: A Controlled Clinical Investigation. Scand. J. Urol..

[16] Antolak S.J. (2024). The Pudendal Syndrome: A Photo Essay of Nerve Compression Damage Visualized at Neurolysis in Patients with Chronic Neuropathic Pelvic Pain. Neurourol. Urodyn..

[17] Yosef A., Ahmed A.G., Al-Hussaini T., Abdellah M.S., Cua G., Bedaiwy M.A. (2016). Chronic Pelvic Pain: Pathogenesis and Validated Assessment. Middle East. Fertil. Soc. J..

[18] Robert R., Prat-Pradal D., Labat J.J., Bensignor M., Raoul S., Rebai R., Leborgne J., Lardoux M.C., Thiodet J. (1998). Anatomic Basis of Chronic Perineal Pain: Role of the Pudendal Nerve. Surg. Radiol. Anat..

[19] Manchikanti L., Singh V., Falco F.J., Kaye A.D., Soin A., Hirsch J.A. (2024). Essentials of Interventional Techniques in Managing Chronic Pain.

[20] Trescot A. (2024). Nerve Blocks for Pelvic Pain. Essentials of Interventional Techniques in Managing Chronic Pain.

[21] Briley J.D., Keenihan E.K., Mathews K.G., Chiavaccini L. (2022). Development of an Ultrasound-Guided Transgluteal Injection of the Pudendal Nerve in Cats: A Cadaveric Study. Vet. Anaesth. Analg..

[22] Levin D., Florcke V., Schmitt D., Kendall M., Patel L.K., Catarci S., Levin D., Van Florcke D., Schmitt M., Kurzava Kendall L. (2024). Fluoroscopy-Guided Transgluteal Pudendal Nerve Block for Pudendal Neuralgia: A Retrospective Case Series. J. Clin. Med..

[23] Murer S., Polidori G., Beaumont F., Bogard F., Polidori É., Kinne M. (2021). Advances in the Therapeutic Approach of Pudendal Neuralgia: A Systematic Review. J. Osteopath. Med..

[24] Avellanal M., Ferreiro A., Diaz-Reganon G., Orts A., Gonzalez-Montero L. (2015). Neuralgia Del Pudendo: Algoritmo de Manejo Diagnóstico y Terapéutico Desde Una Unidad Del Dolor. Prog. Obstet. Ginecol..

[25] Luesma M.J., Galé I., Fernando J. (2021). Diagnostic and Therapeutic Algorithm for Pudendal Nerve Entrapment Syndrome. Med. Clin..

[26] Alfonso Reyes V., Marquet Palomer R. (2016). Pudendal Nerve Entrapment Syndrome. Reflections on Chronic Perineal Pain. FMC.

[27] Dickenson A. (2013). The Neurobiology of Chronic Pain States. Anaesth. Intensive Care Med..

[28] Zhuo M., Wu G., Wu L.J. (2011). Neuronal and Microglial Mechanisms of Neuropathic Pain. Mol. Brain.

[29] Fong A., Schug S.A. (2014). Pathophysiology of Pain: A Practical Primer. Plast. Reconstr. Surg..

[30] Ospelnikova T.P., Shitova A.D., Voskresenskaya O.N., Ermilova E.V. (2023). Neuroinflammation in the Pathogenesis of Neuropathic Pain Syndrome. Neurosci. Behav. Physiol..

[31] Gilron I., Baron R., Jensen T. (2015). Neuropathic Pain: Principles of Diagnosis and Treatment. Mayo Clin. Proc..

[32] Kalso E., Aldington D.J., Moore R.A. (2013). Drugs for Neuropathic Pain. BMJ.

[33] Haanpää M.L., Gourlay G.K., Kent J.L., Miaskowski C., Raja S.N., Schmader K.E., Wells C.D. (2010). Treatment Considerations for Patients With Neuropathic Pain and Other Medical Comorbidities. Mayo Clin. Proc..

[34] Angerer S., Waibel C., Stummer H. (2019). Discrimination in Health Care: A Field Experiment on the Impact of Patients’ Socioeconomic Status on Access to Care. Am. J. Health Econ..

[35] Kanazi G., Johnson R., Dworkin R. (2000). TREATMENT OF POSTHERPETIC NEURALGIA—AN UPDATE. J. Peripher. Nerv. Syst..

[36] Lynch M.E., Campbell F., Clark A.J., Dunbar M.J., Goldstein D., Peng P., Stinson J., Tupper H. (2008). A Systematic Review of the Effect of Waiting for Treatment for Chronic Pain. Pain.

[37] Levesque A., Bautrant E., Quistrebert V., Valancogne G., Riant T., Beer Gabel M., Leroi A.-M., Jottard K., Vancaillie T., Leveque C. (2022). Recommendations on the Management of Pudendal Nerve Entrapment Syndrome: A Formalised Expert Consensus. Eur. J. Pain.

[38] Robert R., Labat J.J., Bensignor M., Glemain P., Deschamps C., Raoul S., Hamel O. (2005). Decompression and Transposition of the Pudendal Nerve in Pudendal Neuralgia: A Randomized Controlled Trial and Long-Term Evaluation. Eur. Urol..

[39] Tsuda M. (2018). Microglia in the CNS and Neuropathic Pain. Adv. Exp. Med. Biol..

[40] Gilron I., Watson C.P.N., Cahill C.M., Moulin D.E. (2006). Neuropathic Pain: A Practical Guide for the Clinician. CMAJ.

[41] Andiman S.E., Maron J.S., Dandolu V., Drugge E., Cosgro R.P., Vasey M.A.M., Spaulding G.C., Ricciardi F., Yaskhi G., Toal C.A. (2024). Impact of Treatment of Pudendal Neuralgia on Pain: A Systematic Review and Meta-Analysis. Int. Urogynecol. J..

[42] Sator-Katzenschlager S.M., Scharbert G., Kress H.G., Frickey N., Ellend A., Gleiss A., Kozek-Langenecker S.A. (2005). Chronic Pelvic Pain Treated with Gabapentin and Amitriptyline: A Randomized Controlled Pilot Study. Wien. Klin. Wochenschr..

[43] McGrath P.A. (1994). Psychological Aspects of Pain Perception. Arch. Oral. Biol..

[44] Conejero I., Olié E., Calati R., Ducasse D., Courtet P. (2018). Psychological Pain, Depression, and Suicide: Recent Evidences and Future Directions. Curr. Psychiatry Rep..

